# Leaf infiltration in plant science: old method, new possibilities

**DOI:** 10.1186/s13007-021-00782-x

**Published:** 2021-07-28

**Authors:** Izabela Anna Chincinska

**Affiliations:** Department of Plant Physiology and Biotechnology, Faculty of Biology, University of Gdańsk, ul. Wita Stwosza 59, 80-308 Gdańsk, Poland

**Keywords:** Leaf infiltration strategies, Syringe infiltration, Vacuum infiltration, Apoplast, Secretome, Phyllosphere, Agroinfiltration, Molecular farming, Infiltration in food industry, Nanomolecules

## Abstract

The penetration of substances from the surface to deep inside plant tissues is called infiltration. Although various plant tissues may be effectively saturated with externally applied fluid, most described infiltration strategies have been developed for leaves. The infiltration process can be spontaneous (under normal atmospheric pressure) or forced by a pressure difference generated between the lamina surface and the inside of the leaf. Spontaneous infiltration of leaf laminae is possible with the use of liquids with sufficiently low surface tension. Forced infiltration is most commonly performed using needle-less syringes or vacuum pumps.

Leaf infiltration is widely used in plant sciences for both research and application purposes, usually as a starting technique to obtain plant material for advanced experimental procedures. Leaf infiltration followed by gentle centrifugation allows to obtain the apoplastic fluid for further analyses including various omics. In studies of plant-microorganism interactions, infiltration is used for the controlled introduction of bacterial suspensions into leaf tissues or for the isolation of microorganisms inhabiting apoplastic spaces of leaves. The methods based on infiltration of target tissues allow the penetration of dyes, fixatives and other substances improving the quality of microscopic imaging. Infiltration has found a special application in plant biotechnology as a method of transient transformation with the use of *Agrobacterium* suspension (agroinfiltration) enabling genetic modifications of mature plant leaves, including the local induction of mutations using genome editing tools. In plant nanobiotechnology, the leaves of the target plants can be infiltrated with suitably prepared nanoparticles, which can act as light sensors or increase the plant resistance to environmental stress. In addition the infiltration has been also intensively studied due to the undesirable effects of this phenomenon in some food technology sectors, such as accidental contamination of leafy greens with pathogenic bacteria during the vacuum cooling process.

This review, inspired by the growing interest of the scientists from various fields of plant science in the phenomenon of infiltration, provides the description of different infiltration methods and summarizes the recent applications of this technique in plant physiology, phytopathology and plant (nano-)biotechnology.

## Introduction

**Fig. 1:**
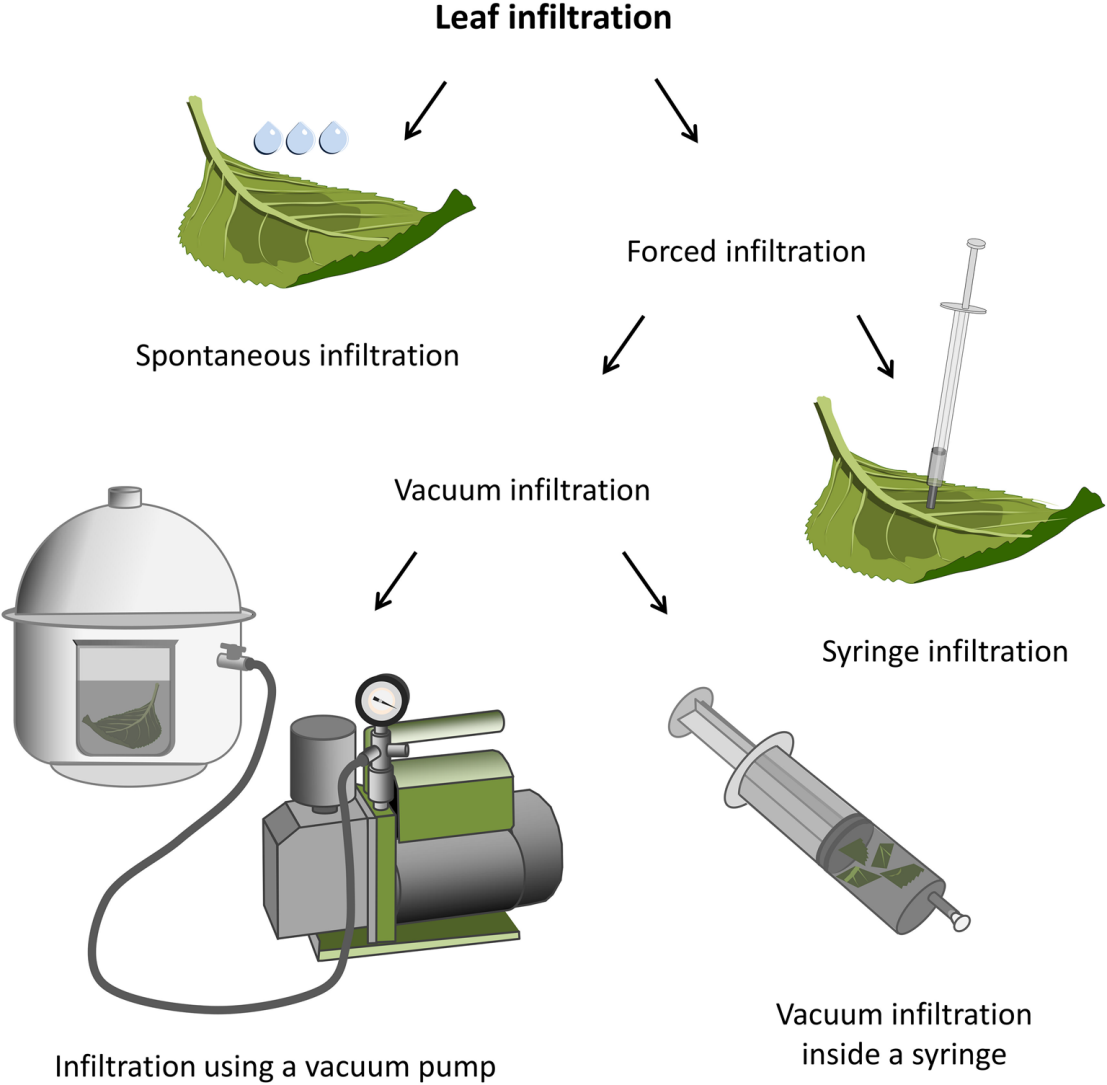
Techniques of leaf infiltration divided according to the pressure used. Spontaneous infiltration is possible under normal atmospheric pressure if the infiltration fluids have sufficiently low surface tension and viscosity. Forced infiltration is driven by the pressure difference generated between the leaf surface and its intercellular spaces. The pressure difference can be generated by exposing target tissues to the vacuum conditions. For this purpose, a vacuum chamber with a pump connected to it can be used, or a suitably large syringe in which the infiltrated leaves or their fragments are placed and then subjected to the vacuum by pulling on the plunger. The infiltration fluid can also be directly pressed inside the leaf using a needleless syringe, the outlet of which is applied closely to the leaf surface

The intercellular spaces in leaf mesophyll play a pivotal role in gas exchange and transpiration. Natural access to them is possible thanks to stomata on the leaf surfaces [1–3]. Stomata are used to reach the intercellular spaces by pathogenic and non-pathogenic microorganisms which will inhabit the leaf intercellular space [4, 5]. Access to internal leaf tissues through the stomata also allows for the experimental application of various types of liquid substances from the outside. This procedure is commonly referred to as leaf infiltration [1, 6, 7]. Infiltration can occur spontaneously or as a forced process in which penetration of substances into the intercellular spaces is driven by the pressure difference between the surface and the inside of the leaf (Fig. 1). During the infiltration process, the gases present in the intercellular space are displaced by the applied liquid. Based on the number of scientific reports in which infiltration was described as one of the research techniques contributing to a larger experiment, it can be concluded that forced infiltration techniques are used more often than spontaneous techniques. Thus the term “infiltration” stands currently for the forced process in which different liquid substances are introduced into the plant tissue using equipment that generates a pressure gradient, such as a needle-less syringe (syringe infiltration) or a vacuum pump (vacuum infiltration) [8–10].

Plant infiltration techniques are useful in various fields of plant science, ranging from plant physiology, molecular physiology and biochemistry through biotechnology to applied plant sciences, such as plant cultivation, horticulture and agriculture [8, 11–14]. Therefore, it appears that the application of various substances to the inside of the leaf tissues using infiltration techniques is carried out by researchers from seemingly disparate fields of plant biology to achieve various research goals. This review aims to bring together the methods used in plant biology in which infiltration is the starting procedure for further, sometimes very advanced research with the use of modern laboratory equipment and the latest molecular technology. Due to the large variety of infiltration applications described in the latest literature and the expanding knowledge about its mechanisms, I have tried to classify and organize the information available on this subject. In the following sections I quote selected examples of research in which the procedure of application of liquid substances to the internal leaf tissue was introduced as a key preliminary procedure for larger experiments. This article also aims to inspire the development of novel leaf infiltration techniques and to search for new applications especially by those plant researchers who have not used infiltration in their work so far.

## Strategies of leaf infiltration

The short term “infiltration” was originally used to describe the phenomenon of spontaneous penetration of liquids through the stomata into the intercellular spaces connected with stomatal cavities [7, 15]. In the past, the spontaneous infiltration phenomenon was used to study stomatal functions, especially for the analysis of stomatal aperture and mechanisms of their control. The idea of using infiltration to study of stomata was first described at the beginning of the 20th century in two German-language publications [7, 16]. In the brief version, the technique was based on treating the leaves of various plant species with low surface tension liquids such as ethanol, xylene, turpentine oil or benzene, which showed different rates of spontaneous penetration into leaf tissues. While almost fully open stomata were required for alcohol spontaneous infiltration, benzene could penetrate even the very slightly open stomata, which was clearly shown for several dozen of the plant species tested [7]. In the decades that followed, the use of infiltration to study stomata has been modified and refined many times by various researchers [17, 18]. A particularly important modification was the replacement of the previously mentioned toxic substances by solutions with less destructive effects on the plant tissue [18]. Currently, those methods have rather historical value, because the study of stomatal functions is performed primarily using direct approaches, including modern microscopic techniques [19, 20]. However, the experience gained over time and the results describing the mechanisms of spontaneous infiltration are still used in plant sciences, especially for practical applications. One of the examples is the use of non-toxic, non-fluorescent substances: perfluorocarbon (PFC), perfluorodecalin (PFD) and perfluoroperhydrophenanthrene (PP11) which improve the quality of in vivo plant leaf imaging, using for example laser scanning confocal microscopy (LSCM), two-photon fluorescence microscopy, second harmonic generation microscopy, and stimulated Raman scattering (SRS) microscopy [21]. Surface tensions of PFC, PFD and PP11 are significantly lower than the tension required to passively overcome the stomatal barrier, therefore they can be used for spontaneous infiltration of leaf tissue [17, 21]. During infiltration, the gases from the apoplastic space of the mesophyll are replaced by PFC, PFD or PP11 to create a homogeneous, undetectable filling of the leaf airspaces resulting in reduced noise and quantifiably clearer images [21]. The results of studies on the mechanisms of spontaneous infiltration are of particular use also when larger areas are subjected to foliar spraying with fertilizer, herbicides, plant protection products or growth regulators [22–26]. Spontaneous infiltration was recently used for introduction of cerium oxide nanoparticles (nanoceria) complexed with the surfactant Silwet L-77 (0.05%) into cotton leaves, improving the salt tolerance of cotton plants [27].

For forced infiltration (Fig. 1), equipment that generates pressure changes is used to introduce fluid into the intercellular space. The simplest tool used for this purpose is a needle-free syringe. The inlet of a syringe filled with the appropriate liquid medium is placed directly on the surface of the leaf lamina, most commonly on the lower (abaxial) side due to the higher abundance of stomata, then the liquid is pressed into the leaf [1, 28]. For larger-scale experiments, forced infiltration using a vacuum pump is a more suitable method. In contrast to syringe infiltration, the method using vacuum pomp is automated and therefore easier to control and more repeatable. Besides, the use of a vacuum pump allows infiltration of various organs and even entire plants. Plant tissues, immersed in the infiltration liquid, are placed in a vacuum chamber and subjected to reduced pressure. In the first phase of infiltration, lowering the pressure in the chamber leads to the release of gases from the stomatal cavities and the adjacent mesophyll airspaces through stomata and possibly through wounding sites [29]. Thus, the pressure within the leaf is lowered with a rate depending on the permeability of the mesophyll and the stomata opening to the gas flow [14]. Then, in the second phase of the process, the plant tissue undergoes rapid re-pressurization, during which the infiltration fluid is drawn into the intercellular space [30].

For very small plants, seedlings, small leaves or leaf fragments, a strategy in which the target tissues are placed together with the infiltration fluid inside a large syringe can also be applied. Pulling the plunger of the syringe, while blocking its opening, generates the negative pressure in the syringe chamber and in the infiltrated tissues. After unblocking the syringe opening and releasing the plunger, the pressure in the tissues is dynamically compensated by drawing the surrounding fluid inside the intercellular spaces [2, 6]. The mechanism of such infiltration is similar to infiltration using a vacuum pump. Thus, the method is a simplified version of vacuum infiltration that can be carried out without access to a pump and a vacuum vessel [2, 6]. Therefore, in the literature, both infiltration methods, driven by a vacuum pump and by a vacuum generated inside a syringe, are referred as “vacuum infiltration” [2, 6]. By contrast, the term "syringe infiltration" (often called simply "infiltration") refers to a method in which the infiltration fluid is forced directly into the leaf lamina from a needleless syringe [10, 28, 31, 32].

In the case of syringe infiltration, the individual skills of the infiltrator and the parameters of the equipment used are of great importance. Syringe infiltration can cause mechanical stress, generating defence reactions in the plant organism which may interfere with or prevent the correct interpretation of the experimental results [9, 28]. Syringe infiltration is also less useful for large-scale application than vacuum infiltration because the method is laborious and time-consuming. Therefore, vacuum infiltration remains more frequently used than syringe infiltration due to its repeatability, the possibility of regulating the pressure value and its duration at each stage of infiltration [10]. Nevertheless, when precise local application of liquids inside the leaf is required, or when various substances need to be applied to multiple leaves on the mother plant, syringe infiltration is a more useful technique.

## Infiltration effectivity and monitoring methods

**Fig. 2:**
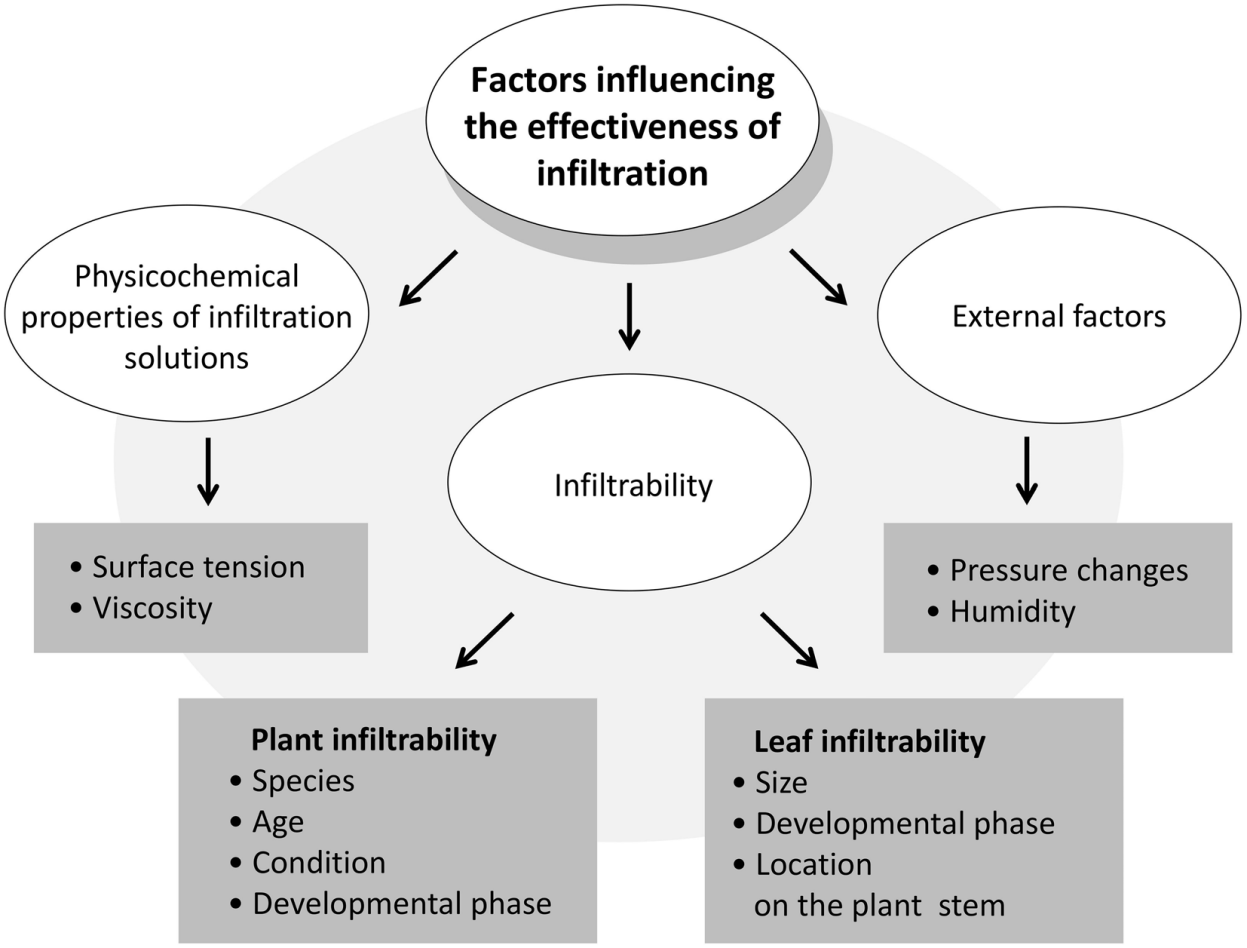
Factors influencing the efficiency of leaf infiltration. All the factors that determine the effectiveness of leaf infiltration can be divided into three groups: 1. Physicochemical properties of an infiltration fluid, 2. Plant and leaf individual susceptibility to infiltration called infiltrability and 3. External factors. For each of these groups, the most important examples of factors are listed. If necessary, the poor infiltrability of plants and their individual leaves can be compensated by optimizing the composition of the infiltration fluid (group 1. factors) and the conditions of the infiltration period (group 3. factors)

The control of infiltration conditions was the subject of numerous studies and various reviews [1, 6, 8, 32]. The factors influencing the effectiveness of infiltration can be divided into three groups: physicochemical properties of infiltration solutions, individual susceptibility of the infiltrated tissue or so-called infiltrability [6], and environmental (external) factors (Fig. 2).

Among the physicochemical properties of the infiltration solution which facilitate its penetration into plant tissues, the most frequently mentioned are surface tension and viscosity. Increasing in the value of surface tension adversely affects the effectiveness of infiltration. Liquids with high surface tension, including pure water, do not penetrate the inner leaf tissue without externally applied pressure [7, 17]. The critical surface tension of 25–30 mN/m was determined experimentally for a model plant *Zebrina purpusii* by using a series of surfactant solutions of different surface tensions. It was shown that only fluids with surface tension below the critical value were able to spontaneously penetrate the stomata [17]. The addition of a surfactant is one of the ways to reduce the surface tension of the infiltration fluid. However, it is important that the final surfactant content is not higher than the critical micelle concentration (CMC). Otherwise, the viscosity of the infiltration fluid is increased [17, 33]. The excessive fluid viscosity is also detrimental to the efficiency of infiltration [18, 26, 33]. This parameter is especially important for the infiltration of bacterial suspensions, because during their growth, the viscosity of the medium increases significantly due to excessive synthesis of exopolysaccharides [34]. The viscosity of bacterial suspensions can lowered by diluting the culture and/or replacing the growth medium with an appropriate infiltration buffer, most often containing MES with the addition of magnesium salt and optional surfactants [32, 35, 36].

Leaf infiltrability is dependent on its individual properties and generally is influenced by factors that affect the size, number and degree of opening of the stomata (thus infiltrability is subject to diurnal control), the chemical composition of the cutin and mesophyll resistance [2, 6, 17, 28]. The parameters characterizing leaf infiltrability directly affect the value of the critical surface tension [17, 18]. Many studies have confirmed that differences in infiltrability are related to plant species, age, condition, developmental phase, and are also largely dependent on the parameters that describe the individual leaves including leaf size, developmental phase or location on the plant stem [6, 28].

The most frequently discussed external factors influencing the infiltration process are pressure changes and humidity. In the first phase of vacuum infiltration a negative pressure is applied (the vacuum values between 20 and 25 kPa are often used) [6, 37]. During this stage, the pressure inside the leaf tissues decreases from atmospheric level to the target pressure at a rate depending on the permeability of the mesophyll as well as the resistance of the stomata to the gas flow [14]. Extending the duration of the first phase has a positive effect on the efficiency of infiltration until the pressure inside the infiltrated tissue reaches a value close to the applied external pressure [14, 29]. For *Lactuca sativa valmaine*, the substomatal cavity pressure reaches the target value after approximately 80, 100 and 120 s with the applied vacuum of 45, 25 and 5 kPa, respectively [29]. Comparable results have been obtained by analyzing the mechanistic models for infiltration [14]. After reaching the target pressure value further extension of the duration of the first phase does not significantly affect the effectiveness of infiltration [14, 29]. During the re-pressurization phase, fluid penetrates into the intercellular spaces of the target tissues. The greater the pressure gradient between the inner spaces of the leaf and its outer surroundings, the deeper the penetration of the infiltration fluid and the faster the saturation of leaf airspaces [14, 29]. Simmons et al. demonstrated that the mass of liquid applied to leaves *of L. sativa* increased proportionally with vacuum intensity. Using a vacuum of 60, 40, 20 and 5 kPa resulted in infiltrating approximately 0.2, 0.3, 0.4 and 0.5 g of bacterial suspension per g of leaf tissue, respectively [29]. Also, the duration of the re-pressurization phase is very important for efficient infiltration. It was shown that during the re-pressurization from 0.7 to 100 kPa for 1 s, the amount of water that penetrated into the target tissue was almost 20% less than the amount of water when the re-pressurization time was 10 s [14]. Extending the re-pressurization time also increased the amount of bacteria that penetrated into the target tissue and the depth of their infiltration thus consequently, positively influenced the level of ectopic expression in the case of agroinfiltration [14, 38, 39].

The importance of moisture for the course of infiltration is interpreted ambiguously. On the one hand, it was suggested that low initial moisture content of the leaf leads to a stronger capillary diffusion of water in the vacuum infiltrated leaf tissues [14]. On the other hand, it is known that well-hydrated plants have open stomata and are much more susceptible to absorbing infiltration fluid forced into the leaf tissues using a needle-free syringe [28]. Due to the opening of the stomata, the optimal water content in the leaf tissues seems to be of fundamental importance for the optimization of the infiltration procedure [28]. Another important aspect is to maintain optimal humidity during the incubation of the leaves after infiltration. Depending on the experimental needs, immediately after infiltration the leaves can be subjected to reduced humidity to evaporate water from infiltration fluids, or increased humidity to stop the transpiration process [28, 40].

Various methods are performed to monitor the effectiveness of leaf infiltration. The simplest is visual evaluation. Infiltrated leaf tissues turn dark green at the site of the infiltration and the size of the dark spot depends on the leaf infiltrability [6, 28]. It is relatively easy to recognize infiltration effects in the case of the vacuum infiltration strategy in which target explants become completely submerged in the fluid because the leaves or their fragments change colour when completely infiltrated with liquid and spontaneously sink into the solution [6]. The air spaces in tissue saturated with liquid are partly eliminated and reflected less light than non-infiltrated tissues, therefore, comparison of reflectivity of the infiltrated with the non-infiltrated leaves has also been proposed for evaluation of infiltration effectivity [41]. However, the most common way to monitor infiltration efficiency is to compare the leaf weight before and after infiltration. The increase in leaf weight as a result of saturation of air spaces with liquid enables assessment of the effectiveness of this process [6, 28, 29]. In the case of more advanced studies aimed at developing new infiltration methods or optimizing the infiltration conditions for plant species known for their poor infiltrability, various more technically advanced methods are recommended to direct observation of tissues impregnated with infiltration solutions. For this purpose, microscopic imaging or X-ray microtomography can be effectively used [39, 42].

## Infiltration in apoplast studies and in secretomics

In secretome studies, especially in secretome proteomics, the isolation of good-quality apoplastic fluid is of particular importance [11, 43]. The most widely used procedure for this purpose is the infiltration-centrifugation method. The technique is amenable for the determination of metabolites and ions in the leaf apoplast and analysis of the plant secretome [6, 44]. In this method, water or a suitably selected buffer is infiltrated into leaf tissue, then the whole leaves or large fragments of leaf are gently centrifuged to recover the so-called apoplast washing fluid (AWF) resulting from the dilution of the apoplastic fluid with infiltration medium [2, 6, 44]. The infiltration-centrifugation technique (in the case of vacuum **i**nfiltration-centrifugation the abbreviation VIC is usually used) allows for quick and easy collection of AWF from leaves. By optimizing the parameters of infiltration and centrifugation to individual needs and adjusting the appropriate infiltration buffer, it is possible to obtain good quality samples containing a representative composition of apoplastic fluid from intact leaves [2, 6, 44, 45]. The infiltration-centrifugation conditions can be adapted for applying the method to various plant species [6].

The AWF isolation method using the infiltration-centrifugation technique is gentle enough to allow minimal contamination of samples by damaged cells. To obtain the contamination-free apoplastic fluid samples, short infiltration times and low centrifugation forces are adjusted individually for target plant material [2, 6]. In optimizing the procedure, various methods for detection of cytoplasmic contaminants in the collected AWF are used. For this purpose, measurements of the activity of marker enzymes such as malate dehydrogenase (MDH), glucose-phosphate isomerase (GPI) and glucose-6-phosphate dehydrogenase (G6PDH), determination of the level of metabolites such as glucose-6-phosphate (G-6-P), or immunodetection of cytoplasmic proteins including MDH, RuBisCo or ATPase are commonly used [2, 46, 47].

Another important parameter for assessing the AWF quality is the dilution factor because the infiltration-centrifugation process leads to dilution of the apoplastic fluid [2]. Estimation of the dilution factor value is necessary to calculate the original concentrations of metabolites in the apoplastic fluid or when experiments requires the AWF concentration at full strength thus matching in vivo metabolite concentrations as accurately as possible, for example when AWF is used as an apoplast-mimicking growth medium for microbes [2]. For determining the AWF dilution factor, markers which are not absorbed, transported or modified after incorporation into the apoplast are used. An example is indigo carmine, which is added to the infiltration medium and measured spectrophotometrically in the obtained AWF at 610 nm [2]. Other substances such as blue dextran or [^14^C] sorbitol have also been used as AWF dilution markers [2, 6, 48].

The use of the infiltration-centrifugation method for apoplast studies has many advantages. One of the most important is that the method allows research on plants growing in natural or close to natural conditions. This is extremely important because the composition of the plant secretome depends on biotic and abiotic environmental conditions [46, 49, 50]. Such studies are therefore not possible using other secretome isolation techniques such as those in which the proteins released into the extracellular space are isolated from conditioned (post-culture) media derived from hydroponic or plant cell cultures [51]. Therefore, the application of the infiltration-centrifugation for secretome sampling overcomes the inconveniences related to conducting plant cultures in vitro. An interesting aspect is the possibility of isolating plant secretome in response to climate change and/or environmental pollution using infiltration-centrifugation. This methodology was applied in a study of the detrimental effect of tropospheric ozone (O_3_) on various plant species, showing significant protection of cells from O_3_ by ascorbate in leaf apoplast [52, 53]. In addition, using the infiltration-centrifugation method, secretome samples can be collected from large plants such as trees, or from species whose populations grow in large areas. It is most useful in studies of plants that occur naturally in particularly demanding, complicated environmental conditions where it is not possible to transfer the total research of such plants to the laboratory without simultaneously generating changes in the secretome.

The isolation of AWF by the infiltration-centrifugation method may also be useful for the analysis of recombinant proteins secreted into the apoplast of transgenic plants [54–56]. The effective isolation and purification of recombinant proteins from tissue of transgenic plants is one of the most critical points in developing plant expression systems as this step is costly and time-consuming [57, 58]. Obtaining a pure recombinant product is crucial due to its biotherapeutic purpose and for planning its further applications [57, 58]. One of the most frequently used approaches towards increasing the efficiency of recombinant protein production is targeting them to various subcellular structures, including the apoplast [54–56, 59]. Proteins dissolved in the apoplastic fluid are much easier to extract and a higher yield can be obtained [2, 54]. The use of the infiltration-centrifugation method has been recently proposed for the isolation of human recombinant DNase I secreted into the apoplast of transgenic tobacco and *Luffa cylindrica* plants [54].

## Research of plant-microorganism relationships

The surface of above‐ground organs of plants, known as the phyllosphere, is the habitat of numerous microorganisms [60, 61]. The microbiome associated with the leaf phyllosphere is currently the subject of intensive research in various fields of plant biology and microbiology [62, 63]. Leaf infiltration is widely used in the research of plant disease mechanisms, where it has been developed for the controlled induction of plant infections, for example with the model prokaryotic pathogen *Pseudomonas syringae*, under standard laboratory conditions [4, 5, 64]. Although various bacteria and not only phytopathogens can actively penetrate the internal leaf tissues via stomata or tissue wounds, applying the inoculum to the leaf surface for spontaneous infiltration of microorganisms is a less efficient method than forced infiltration [20, 47, 65, 66]. Due to the need for spatially precise application of the inoculum and control of microbial load introduced into the plant tissue, it is preferable to use syringe infiltration for this purpose rather than vacuum infiltration [4, 5, 12].

Leaf intercellular spaces, due to the presence of many nutritional compounds, optimal humidity and protection against the direct impact of external environmental conditions, provide unique habitats for various microorganisms, many of which are endophytes that are not considered pathogenic [4, 66]. The colonization of plant organisms by bacterial or fungal endophytes can enhance resistance to biotic and abiotic stress, supporting the healthy growth and development of the plant hosts [12, 67]. For this reason, numerous attempts have recently been made to involve these endophytic microorganisms in the design of modern environmentally-friendly plant protection products [68, 69]. Spontaneous and forced infiltration methods can be useful in the design and testing of bio-protection products containing microbes with an antagonistic effect on phytopathogens. These two infiltration techniques were used, for example, to test the protective effect of the insect pathogen *Pseudomonas entomophila* naturally inhabiting the soil, as it displayed a strong activity against the phytopathogenic bacteria *Xanthomonas citri* subsp *citri* (*Xcc*), the etiological agent of citrus canker disease [66]. Applying the mixture of *P. entomophila* and *Xcc* to the leaves using forced and spontaneous infiltration led to a significant reduction in the number of canker lesions in highly susceptible citrus leaves [66].

Studies of the microbes colonizing leaves are also of great importance in the field of bacteriological safety in the food industry [63, 70]. It has been shown, for example, that although the enteric pathogen *Salmonella enterica,* is not a native plant endophyte, can actively infiltrate the deep leaf structures and multiply there [47]. Studying the diversity of microorganisms in endophytic communities and explaining the interactions between them can help to develop a strategy of the bacteriological protection of edible plants against their colonization with human pathogens. Another important reason for the study of leaf-phyllosphere microorganisms is their potential with regard to air purification from pollutants from anthropogenic and natural sources (phylloremediation) [63]. For the leaf microbiome studies, infiltration methods are used for example in experiments involving the introduction of various substances, including microorganism suspensions, into the apoplastic space to follow specific plant reactions or modifying their functions in a controlled manner [12]. Additionally, the infiltration-centrifugation method can be used to the extraction of AWF added to media prepared for endophyte microorganism cultured in vitro [2, 12].

Hong et al. emphasized the need for a detailed understanding of the molecular basis of the interactions between endophytes, phytopathogens and host plants. It seems particularly interesting to study the impact of these tripartite relationships on overall plant health [12]. Of particular importance for this type of research are those experiments which enable the tracking of these relationships in the natural environment in intact leaves. In particular, the technique has been useful in illustrating the occurrence of qualitative and quantitative differences in the plant microbiome depending on the conditions under which plants are grown [4, 63]. The AWF extraction from leaves infiltrated with appropriate buffers can significantly improve this type of research. The infiltration-centrifugation method was used for the isolation of four novel endophytic bacteria from *Arabidopsis thaliana* [12]. In this experiment, plants were inoculated with *Pseudomonas syringae* pv. *tomato* DC3000 by syringe-infiltration, and a few days later the bacteria were isolated from the AWF derived from the upper uninfected leaves. Among the isolated microbes were members of *Rhodococcus* species that have never before been identified as leaf-inhabiting endophytes [12]. Further studies showed that the bacterial strains isolated from AWF can proliferate intercellularly in the leaf tissues of *A. thaliana* grown under sterile conditions. It has also been shown that the endophytic bacteria exhibit antagonistic activity against various phytopathogens, including *Pseudomonas syringae* pv. *tomato* DC3000 and *Fusarium oxysporum* pv. conglutinans [12].

Properly collected AWF contains at most only traces of cell contamination, so it is worth considering the use of infiltration-centrifugation as an attractive alternative to invasive methods of plant tissue homogenization used to study microorganisms inhabiting internal leaf tissues [2, 70]. It is worth considering the adaptation of AWF extraction procedures towards using them as a starting material for culture-independent microbiome studies using, for example the Illumina Miseq platform. The material for metagenomic analyses obtained in this way could help to circumvent some of the basic problems encountered when using DNA derived from whole leaves macerated using standard protocols, including the low-throughput nature of plant tissue maceration and the prevalence of plant plastid DNA in metagenomic DNA extracts, which is typically coamplified via PCR strategies that target the bacterial 16S rRNA gene [70]. The method of pure AWF extraction for metagenomic DNA purification and library preparation could be helpful to overcome these problems. In addition, the optimization of the strategy for obtaining genetic material for metagenomic analyses from the AWF may increase the possibility of conducting metagenomic analyses in the environment, and in particular, facilitate the tracking of metagenome changes in real-time.

## Plant transformation

Generation of genetically modified plants with the use of *Agrobacterium* carrying a binary vector containing genes of interest requires the penetration of the bacteria into the target tissues. Commonly, this procedure is carried out using plant in vitro cultures [71–73]. During the co-cultivation step, *Agrobacterium* infiltrates leaves and cotyledons, which are the plant organs most often prepared as explants for transformation [30, 74]. The effectiveness of bacterial suspension penetration into the target tissues can be increased by the use of tools driving forced infiltration. There are many examples of the use of the forced infiltration for improving the effectiveness of stable plant transformation in vitro. This method is of particular importance especially in the case of plant species for which it is difficult to establish a stable transformation protocol [71].

### Agroinfiltration

In plant biotechnology, infiltration is most often associated with the popular and globally-used transient transformation technique of agroinfiltration, in which an *Agrobacterium* suspension is pressed into the target tissue of plants growing *in planta*. Agroinfiltration is widely used to test genetic constructs before they are introduced into stable lines and to study gene activity [75–79] or as a tool for the generation of plant expression platforms (plant biofactories) for recombinant protein production [36, 80–82]. The use of agroinfiltration in the above-mentioned cases allows researchers to bypass the costly and time-consuming in vitro cultures. This is important not only for rapid induction of ectopic expression but also for avoiding the development of a generation of somaclonal variants, the formation of which during in vitro regeneration is problematic for the interpretation of phenotypic effects [78].

The successful introduction of *Agrobacterium* into leaf tissue is considered one of the most important requirements affecting the expression level of the foreign gene [1, 30, 83]. Leaves can be agroinfiltrated directly on the mother plant or after cutting [8, 28]. Optimization of the agroinfiltration process involves the appropriate preparation of plant tissue to increase its infiltrability. Generally, plants intended for agroinfiltration must grow under optimal conditions conducive to the opening of the stomata and the formation of permeable mesophyll tissue. In the case of agroinfiltration of leaves separated from the mother plants (e.g., when leaves for agroinfiltration are collected in an open space from plants growing in different environmental conditions), the leaves can be incubated for a certain time under conditions conducive to the opening of the stomata immediately after harvest and before infiltrating [28]. Some agroinfiltration protocols introduce procedures supporting infiltrability [e.g., Sonication-assisted Agrobacterium-mediated transformation (SAAT)] that were tested previously during standard in vitro agrotransformation [84, 85]. It has been shown that treating tissues with ultrasound just before agroinfiltration can significantly increase the efficiency of this process [35, 84, 86]. In the case of plants that are very resistant to infiltration controlled wounding of target tissues is sometimes carried out, for example by repeated needle puncture of the leaf blade within the area where the infiltration will be carried out [87]. Depending on the target plant species and the bacterial strains used for transformation, optimization of the agroinfiltration procedure generally also requires adjusting the composition of the infiltration buffer and bacterial cell density. The physicochemical properties of an infiltration fluid are often adjusted based on knowledge gained decades ago from research on spontaneous infiltration [17, 32, 35].

The application of agroinfiltration allows for single and multigene transformations. As in the case of stable transformation, multigene constructs may be used for this purpose [88], but in general, it is much simpler to perform a co-infiltration with a mix of *Agrobacterium* strains carrying constructs containing different transgenes [31, 87, 89]. Theoretically, the number of the co-infiltrated constructs is unlimited, because the multigene transformation using agroinfiltration does not require selection markers, which are normally necessary for the regeneration of stably transformed plantlets. This is also a great advantage of agroinfiltration. Another way to obtain multigene expression is a method in which leaves of stable transgenic plants are agroinfiltrated with additional transgenes [90, 91].

In the case of agroinfiltration, there is no possibility of preselecting transformed tissues using antibiotics or herbicides. Despite this, it is possible to obtain a satisfactory level of foreign gene expression after applying appropriate procedures to optimize agroinfiltration [1, 28, 87]. In some experiments, for example, when the ectopically expressed gene leads to a pronounced hypersensitivity reaction [92], the effects of agroinfiltration are directly observable [35, 75, 92]. In turn, the direct microscopic observation of phenotype effects in agroinfiltrated leaves is possible in the subcellular localization tests using fusion constructs with reporter fluorescent proteins [77, 93]. In this case, agroinfiltrated tissue sections can also be treated with cell wall degrading enzymes to obtain protoplasts [31]. The generation of protoplasts from agroinfiltrated tissues very often improves the quality of microscopic imaging and increases the sensitivity of the fluorescent signal detection. Interestingly, it was shown independently of agroinfiltration that infiltration techniques can be also used successfully to optimize protoplast preparation procedures. Introducing solutions containing cellulose and macerozyme directly to the intercellular space of *Phaseolus vulgaris* leaves using a vacuum significantly increased the yield of leaf mesophyll protoplasts [85]. When gene expression analysis using agroinfiltration does not allow the observation of direct phenotypic effects, it is necessary to use various foreign gene detection tools at the molecular level, including sequencing.

### Agroinfiltration in genome editing

In recent years it has been shown that agroinfiltration of various plant species is applicable to test mutations induced at defined loci using various genome editing tools [77, 78, 94]. The use of agroinfiltration for genome editing can be a solution to bypass the problem of the generation of mutant plants in the case of mutations where the target gene proves to be lethal, and thus the regeneration of stably modified plant lines is impossible.

At the moment, the most popular genome-editing tool is the bacterial clustered regularly interspaced short palindromic repeats (CRISPR) system, comprising a CRISPR-associated 9 (Cas9) nuclease and an engineered single guide RNA (sgRNA) that specifies a targeted nucleic acid sequence. Nekrasov et al. described effective coexpression of the GFP-Cas9 gene and sgRNA using an *Arabidopsis* U6 promoter with the 20 bp guide sequence targeting the phytoene desaturase gene (*PDS*) in agroinfiltrated *N. benthamiana.* Molecular analyses confirmed the induction of local mutations in *PDS* of agroinfiltrated tissue. Moreover, it was also shown that it is possible to regenerate plantlets containing mutations in the *PDS* locus from the leaves transiently expressing the *GFP-Cas9* and *sgRNA* [77]. Such plant lines, regenerated from agroinfiltrated leaves, present mostly mosaic or heterozygous genotypes, however, they can be used for the production of seeds and consequently to obtain stable plant lines [77].

Another example of the effective use of agroinfiltration for genome editing *in planta* was described by Ma et al. [78]. Using the transcription activator-like effector nuclease (TALEN), the authors induced local mutations into the polyploid genome of two potato cultivars. Many researchers emphasize the importance of using TALEN as a system capable of generating a less off-target rate than the conventional CRISPR-Cas approach. This is of particular importance for successful gene knockout in polyploid organisms [78].

In both of the cases of gene editing strategy described above, it was not possible to directly observe the phenotypic effects. However, the restriction site mutation assay (RSM, restriction site loss assay) method proved to be useful for the initial detection and pre-selection of mutations in the agroinfiltrated tissues. The use of this molecular method is possible when dealing with the loss of the restriction site that was originally located at the edited gene. The use of the RSM assay allows for pre-scanning of agroinfiltrated tissues in terms of the content of induced mutations [78].

## Infiltration in molecular farming

The production of recombinant proteins using plant organisms as expression systems is called molecular farming. In this rapidly developing field of plant biotechnology, infiltration techniques are used primarily to generate transient modified plant tissues by introducing *Agrobacterium* carrying foreign genes. The agroinfiltrated tissues thus become biofactories to produce recombinant proteins [10, 80–82, 95, 96]. Recombinant proteins are used in many fields of science, in particular in broadly understood medicine. So far, various recombinant proteins, vaccine components, enzymes, blood proteins and monoclonal antibodies have been produced in agroinfiltrated leaves [10, 36, 54, 80, 82, 97, 98].

Many of the recombinant proteins have multimeric structures and/or post-translational modifications necessary for their full activity. The multigene transformation strategies combining stable modification methods and agroinfiltration (co-infiltration using multiple genetic constructs) have gained particular importance in plant glycoengineering [91, 99–101]. The multigene modification enables for example the humanization of glycosylation patterns in the plant-derived recombinant proteins. For this purpose, methods for knockout or reduction of the expression of the native genes corresponding to typically plant post-translational modifications are used. In parallel, genes encoding human enzymes enabling the human-type modification of recombinant proteins are introduced to the plant genome. An example is a successful production of recombinant human immunoglobulin A (IgA) with defined *N*- and *O*-glycans *in planta* [90, 99]*.* Due to the complicated multimeric structure and the presence of *N*- and *O*-glycans, the production of recombinant IgA variants is one of the most difficult challenges in plant biotechnology. The *in planta* production of IgA was successful by combining agroinfiltration with stable transformation methods [56, 90]. For IgA production, glycoengineered *N. benthamiana* ΔXT/FT lines were used, in which the expression of two enzymes responsible for introducing typical plant modifications to the structure of *N*-glycans, the endogenous β1,2-xylosyltransferase (XylT) and α1,3-fucosyltransferase (FucT), was stably reduced using RNA interference (RNAi) technology [56]. The ΔXT/FT lines were infiltrated with a mix of several different *Agrobacterium* strains carrying transgenes encoding the structural polypeptides forming double IgA and, also, proteins that do not build immunoglobulin structures but are relevant for the further posttranslational modifications necessary to obtain fully functional and homogeneous recombinant dimeric IgAs [56, 90].

## Infiltration in plant nanobiotechnology

In recent years, the importance of the use of nanoparticles in various industries has grown, but at the same time, many studies indicate the harmfulness of nanoparticles to the environment [80, 102, 103]. The growing pollution of the environment by nanoparticles and their impact on plants is of serious concern. On the other hand, many research results show the possibility of using nanotechnology in agriculture and the food industry. Studies on both the positive and negative effects of nanoparticles on plants is often carried out in vivo, where one of the proposed methods of controlled application of nanoparticles to plants is foliar infiltration [104]. Leaf infiltration is also a method used to engineer plant nanobionic sensors for detection of environmental pollutants [105, 106].

The use of infiltration techniques for precise and homogeneous application of nanoparticle suspensions to leaf tissues was demonstrated with the example of thioglycolic acid-coated quantum dots (TGA-QD) with a cadmium telluride (CdTe) core and cadmium sulphide (CdS) shell. Due to their small size, versatile surface chemistry, and outstanding optical properties, quantum dots are recognized as an ideal model for plant nanobiotechnology research, especially for the study of nanoparticle uptake, transport, and distribution in plants by confocal microscopy tools [104]. However, the authors also suggest that the presented infiltration delivery methods can be extended to other nanomaterials, such as nanosensors or drug delivery carriers, having at least one dimension smaller than the plant cell wall porosity [104]. An example where the developed leaf infiltration procedure was used in nanobiotechnological research is the experiment in which increased salinity tolerance in *Arabidopsis thaliana* was induced by applying cerium oxide nanoparticles (nanoceria) inside the leaf [107]. The nanoceria exhibit the unique capability of catalytically reducing levels of stress-induced reactive oxygen species including hydroxyl radicals that lack enzymatic scavenging pathways. It was demonstrated that catalytic scavenging of hydroxyl radicals by nanoceria in *A. thaliana* leaves significantly improves mesophyll retention of K^+^ ions, which is crucial for increasing salinity stress tolerance [107].

## Infiltration in the food industry is not always a desirable phenomenon

Recently, plant tissues infiltration mechanisms are being studied not only by researchers whose aim is to improve infiltration effectivity but also in research areas where infiltration is perceived as a very unfavourable process, for example in the food industry dealing with microbial food contamination [14, 20, 70].

Several bacteria, including *Escherichia coli* and *Salmonella enterica*, can infiltrate available openings at the leaf surface, such as stomata, cuts and wounds, penetrating the depths below the leaf epidermis [65]. Such contaminated vegetables are impossible to clean at home, which poses a threat to human health and life. The chemotactic penetration of bacteria into the intercellular spaces of leaves was thoroughly tested using a mechanistic model and confirmed experimentally on selected leaf vegetables. In these studies, light conditions conducive to photosynthesis, the presence of high initial sugar content levels due to pre-exposure of the leaf to light, high chemotactic ability of bacteria, and wide stomatal size were listed as the most important factors supporting bacterial infiltration into leafy vegetables [65].

Recent studies have also shown that vacuum cooling, which is widely used in the food industry, can promote infiltration of various contaminations that may be potentially present on food surfaces, including pathogenic microorganisms, such as norovirus, *Salmonella* and *Escherichia coli* [14, 20].

The vacuum is used as a rapid cooling technique applied especially to fruits, vegetables and in particular leafy greens [20]. The process of vacuum cooling is similar to the vacuum infiltration method used in plant science. During this process, the plant material is placed in a vacuum chamber and sprinkled with water to maintain good condition of the plants. The action of the reduced pressure generates intensive water evaporation resulting in a lower temperature [20]. As in the course of standard vacuum infiltration, during the re-pressurization step of vacuum cooling, water is pushed through the stomatal openings and the leaf mesophyll due to the large pressure gradients created within the leaf section [14]. When the vegetables have surface contaminants, these contaminants infiltrate the plant tissues via the water. From an epidemiological point of view, understanding this phenomenon is necessary for reducing food contamination. Therefore, it is currently being intensively studied and mathematical models have been developed to describe the infiltration process associated with vacuum cooling [14, 20]. The developed models allow for a detailed interpretation of a wide range of factors known to affect the course of the infiltration process and for theoretical prediction of the infiltration efficiency after changing certain parameter [14, 20].

Other helpful tools are the models constructed for the vacuum impregnation process, another technology important for the food industry which is used to facilitate the impregnation of vegetable tissues with different solutions (e.g., solutions containing antioxidants, antimicrobial agents or cryoprotectants) to improve their performance properties or to extend their durability in long-term storage [39, 108, 109]. The described models may be useful in planning, optimizing and controlling the infiltration process to achieve optimal results and repeatability of the planned experiments in the broadly understood fields of plant science.

## Conclusions

**Fig. 3:**
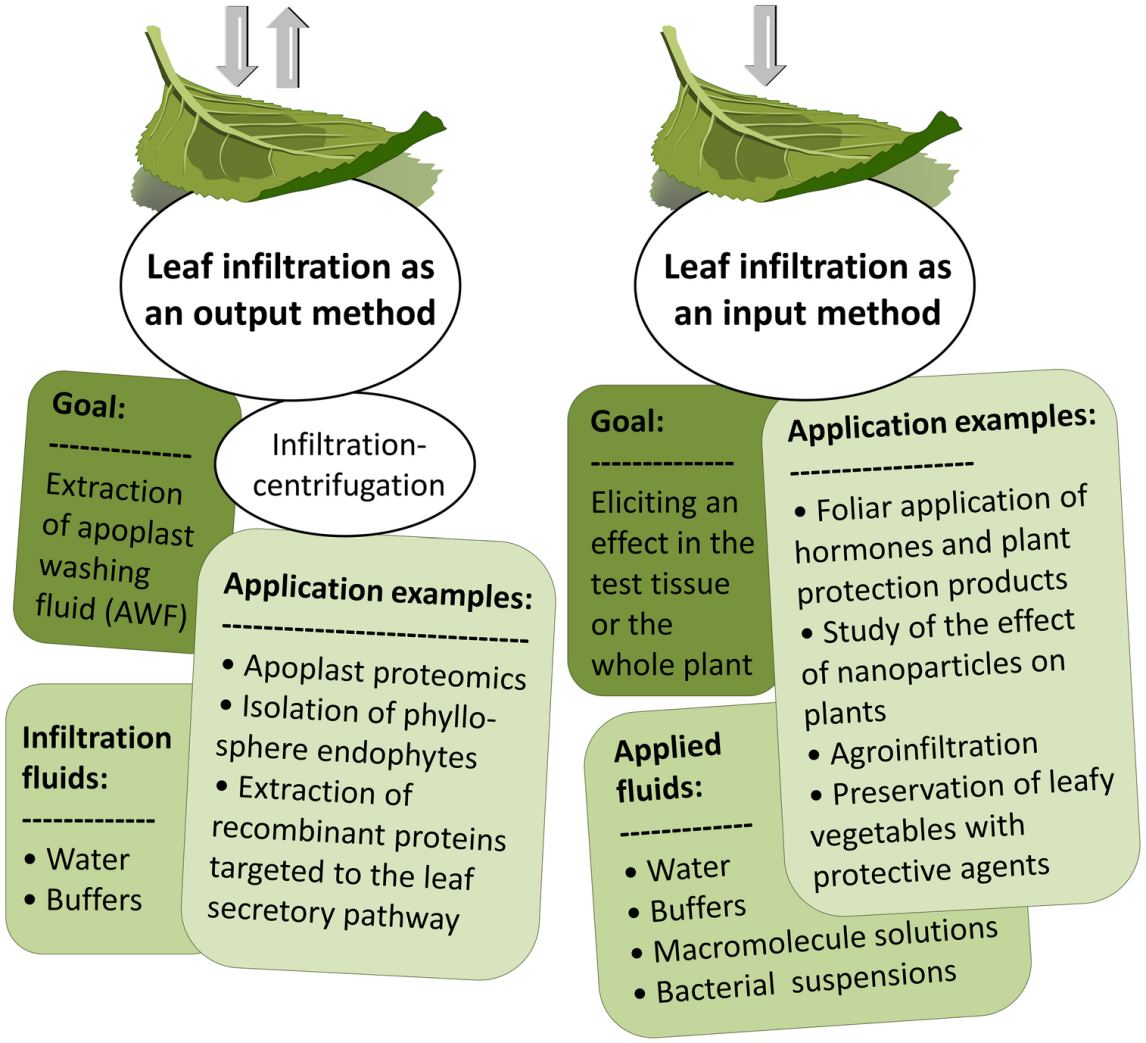
Application of leaf infiltration-based methods in plant sciences. Two categories of experiments can be distinguished: 1. The infiltration as an output method in which leaves or their fragments after infiltration are centrifuged in order to recover the apoplastic fluid. The apoplastic fluid can be subjected to further analyses by a wide range of techniques. 2. The use of infiltration as an output method includes experiments in which the application of various substances to the internal leaf tissues is intended to induce the studied effect, such as hormone application or agroinfiltration

The techniques of leaf infiltration consisting of the application of various liquid substances into the intercellular spaces of the mesophyll through the openings of the stomata are widely used by plant researchers both for cognitive and application purposes (Table 1). On the basis of the examples described in this review, two categories of experiments using leaf infiltration techniques can be distinguished (Fig. 3). The first category includes experiments in which the application of various substances to the internal leaf tissues is intended to induce the studied effect (leaf infiltration as an input method), such as hormone application or agroinfiltration. The another category relates to the experiment aimed at characterization of the plant apoplast (leaf infiltration as an output method). In this case, after infiltration of the intercellular spaces with water or an appropriately selected buffer, the buffer is recovered in the form of fluid containing substances derived from the apoplastic leaf space. The apoplastic fluid obtained by this method can not only be subjected to further analyses by a wide range of omics techniques, but also be used to isolate and cultivate microorganisms inhabiting the intercellular leaf space. The use of infiltration as an output method can also be a way to monitor transgenic plants and to isolate the product of ectopic expression secreted into the apoplast. After studying many of the latest publications describing experiments in which the necessary initial stage was leaf lamina infiltration, it can be safely stated that infiltration is the equivalent of injection, which is the basic method used in scientific laboratories where research is carried out with animal models.

**Table 1:** Summary of various leaf infiltration techniques and their applications

Field of plant science	Application	The purpose of the leaves infiltration	Infiltrated substances	Method used for leaf infiltration/plant material	Selected infiltrated species; additional information^a^	References^b^
	Studies using output infiltration methods					
Molecular plant physiology, apoplast studies, OMICS	Secretome sampling from plants growing in the artificial conditions and (or) in the natural environment	Leaf infiltration is the first step in the infiltration-centrifugation procedure. The method allows the recovery of AWF from the intercellular spaces of plant leaves	Solutions with diverse composition and pH, precisely selected for the experimental needs, e. g., pure water or multi-component buffers containing additional substances such as protease inhibitors in the case of proteomic studies. Markers, such as malate dehydrogenase (MDH), glucose-phosphate isomerase (GPI) or glucose-6-phosphate dehydrogenase (G6PDH), are often added to the infiltration buffer to enable the quality assessment of the isolated AWF	Vacuum infiltration using 50–60 ml syringe or vacuum pump/whole leaves infiltration directly on the mother plant, harvested whole leaves and leaf fragments	*Vitis vinifera *cv. ‘Trincadeira’, *V. vinifera* cv. ‘Regent’	[37]
					*Arabidopsis thaliana*	[44]
					*Prunus persica* cv. Wanxifei	[53]
					*Zea mays*	[45]
					hybrid poplar clone ‘546’: *Populus deltoides *cv. 55/56 × *P. deltoides* cv. Imperial	[52]
					*Faseolus vulgaris, Solanum lycopersicum, A. thaliana*	[2]
					*Oriza sativa*, *Triticum aestivum*, *Phaseolus vulgaris*, *Spinacia oleracea*	[48]
					*Vicia faba, S. oleracea, Beta vulgaris*, *Z. mays*	[6]
Molecular plant physiology, microbiology	Isolation of microorganisms inhabiting the leaf apoplastic spaces or isolation of AWF as a component of media for the cultivation of endophytic microbes				*Lactuca sativa* L. var. *crispa* cv. Salinas—*Salmonella enterica* Serovar Typhimurium	[47]
					*Z. mays—Pantoea stewartii* subsp *stewartii*	[45]
					*A. thaliana—Pseudomonas syringae pv. tomato* DC3000*, S. lycopersicum var. cerasiforme* cv. ‘Tenten’	[11]
Plant biotechnology, molecular pharming	Study of recombinant proteins secreted into the apoplast of transgenic plants		Composition and pH of the infiltration buffer selected for the most effective recovery of recombinant proteins; common components: Tris-NaCl (20–100 mM; pH 5.5–7.7), MgCl_2_ (10 mM), EDTA (2 mM), NaCl (100 mM), NaOAc (20 mM), sodium metabisulphite (4 mM)		*Nicotiana tabacum*—recombinant human deoxyribonuclease I (rhDNaseI)	[54]
					*N. benthamiana*—griffithsin (GRFT)	[92]
					*N. benthamiana*—recombinant IgA with defined N- and O-glycans	[56]
					*N. tabacum*—thermostable xylanase from *Clostridium thermocellum*	[55]
	Studies using input infiltration methods					
Plant—microorganism—environment relationships, phytopathology	Plant-phytopathogen interactions study. Etiology of plant diseases. Investigation of plant defense mechanisms	Leaf infiltration with suspension of phytopathogen cells as method for controlled induction of plant infection	Suspension of phytopathogens or non-pathogen endophyte cells in water or properly selected infiltration medium, most often composed of MES (10 mM) and magnesium salt (MgCl_2_ or MgSO_4_, 10mM).	Spontaneous infiltration, forced infiltration/whole leaves on the mother plant, whole harvested leaves, leaf fragments	*Citrus × limonia*—*Xanthomonas citri* subsp, citri	[66]
					*A. thaliana*—*P. syringae pv. tomato *DC3000	[4]
					*A. thaliana, Solanum lycopersicum* var. cerasiforme cv. ‘Tenten,’—*P. syringae pv. tomato* DC3000	[11]
					*A. thaliana*—*P. syringae* *pv. maculicola* ES4326	[5]
Plant—microorganism—environment relationships, microbial ecology	Leaf microbiome studies, endophyte-plant-phytopathogen tripartite interactions and their importance for the overall health of a plant	Introducing suspension of various microorganisms, e.g., endophytic bacteria into the intercellular leaf spaces			*A. thaliana *- *Blumeria graminis *f. sp. *Hordei*	[44]
					*A. thaliana*, *S. lycopersicum* var. cerasiforme cv. ‘Tenten—*Bacillus cereus* (GU982920.1), *Variovorax paradoxus* (JN990697.1), *Rhodococcus kyotonensis *(AB920569.1)*, R. corynebacteriodies* (AY438619.1)	[11]
Applied microbiology, agricultural microbiology	Design and testing of plant protection products containing microorganism with an antagonistic effect towards phytopathogens				*Citrus × limonia*—*Pseudomonas entomophila, Xanthomonas citri *subsp*, *citri*; Bacillus amyloliquefaciens LE109*	[66]
					*S. lycopersicum var. cerasiforme cv.* *‘*Tenten—*P. syringae pv. tomato DC3000, B. cereus (GU982920.1)*	[11]
Applied microbiology, food technology	Study of the activity of human pathogens in the contamined tissues of edible plants especially leafy vegetables	The cotrolled introduction of human pathogens into the tissues of leafy vegetables			*L. sativa* L. var crispa cv. Salinas—*Salmonella enterica* Serovar Typhimurium	[47]
					*L. sativa* L.—*Escherichia coli *O157: H7 GFPlux	[20]
Plant biotechnology, in vitro cultures	Production of stable transformed plant lines and lines with modified genome	The spontaneous infiltration of leaf explantates with *Agrobacterium* is the first step in the generation of transgenic plants using agrotransformation in vitro. The use of forced infiltration of explantates can additionally increase the effectiveness of the transformation	For spontaneous in vitro infiltration, a suspension of* Agrobacterium* in a culture medium (e.g., YEB) is commonly used (with the addition of acetosyringone (100–200 µM) optionally). In the case of forced infiltration, the A*grobacterium* cells are usually re-suspended in a medium of the same composition as that used for the transient transformation of plants by agroinfiltration	Spontaneous infiltraction, forced infiltration/leaf fragments, cotyledons	*A. thaliana*, *Fragaria vesca *cv. ‘YW5AF7’,* N. benthamiana*—GFP	[9]
					*Malus domestica* cv. ‘Gala’; *Pyrus communis* cv. ‘Conference’—GUS	[71]
					*Taraxacum officinale - *GUS	[72]
					*N. tabacum*—CRISPR-Cas9-mediated knockout of NtFAD2-2	[36]
Plant biotechnology, transient plant transformation using agroinfiltration	Generation of transient transformed leaf tissues or genome edition for functional characterisation of genes in planta	Infiltration/co-infiltration of intercellular leaf spaces with a suspension containing *Agrobacterium* carrying the target genes	*Agrobacterium* suspension in infiltration medium containing MES (10 mM, pH 5.5–5.6), MgSO_4_ or MgCl_2_ (10 mM), and possibly additional substances to increase the transformation efficiency, e.g., acetosyringone (100–150 µM); 5-azacydine AzaC (20 µM), ascorbate acid (0.56 mM), DTT (0.5 mM); surfactants, e.g., Tween 20 (0.015–0.03%), Silwet L-77 (0.01%), Triton™ X (0.001%)	Forced infiltration/whole plants, intact leaves direct on mother plants, whole harvested leaves, leaf fragments	*Populus* clones—GFP, LUC, GUS, CBL1, MTP1, C4H, GT47C, MYB221, and PrxQ, The coding regions of three key activators of secondary cell wall biosynthesis, *Populus davidiana *× *P. bolleana*	[89]
					*Canabis sativa*—GUS; The phytoene desaturase gene was silenced with a transient hairpin RNA expression, resulting in an albino phenotype in the leaves	[35]
					*Sorghum bicolor*, GFP	[87]
					*N. benthamiana*—sucrose transporters StSUT1, StSUT2, StSUT4 and their interaction partner SNARE/VAMP	[31]
					*N. benthamiana*—marker genes under the control of the optogenetic system PULSE	[76]
					*Luffa cylindrica*—GUS	[28]
					*N. benthamiana*—GFP, GUS, mouse granulocyte-macrophage colony-stimulating factor, and human fibroblast growth factor 1	[8]
					*Medicago truncatula *cv. R108, *N. benthamiana*—MYB transcription factors: MtLAP1, a MYB transcription factor involved in the regulation of the anthocyanin pathway, various TF flowering time regulators, AcMYB10 or 35S:AtLEC2	[75]
					*N. benthamiana*—GUS	[32]
					*N. benthamiana*—cooexpression of GFP-Cas9 and sgRNA with the guide sequence within the PDS gene under U6 promoter	[77]
					*S. tuberosum* cv. Russet Burbank, *S. tuberosum* cv. Shepody—TALENs targeted to endogenous starch branching enzyme and an acid invertase	[78]
					*Dendrobium catenatum *Lindl.—GFP, GUS	[36]
					*Glycine max, N. benthamiana*, GUS	[84]
	Generation of transiently transformed leaf tissues or genome edition to create plant bioreactors capable of producing recombinant proteins with potential utility, e.g., in medicine, industry, agriculture				*N. benthamiana* ΔXT/FT—ACE2-Fc fusion protein consisting of the Angiotensin-Converting Enzyme 2 (ACE2)—the primary host cell receptor for SARS-CoV-2 binding and the fragment crystallizable (Fc) of human IgG	[96]
					*N. benthamiana*—capsid protein of hepatitis B virus; HBc	[80]
					*N. benthamiana*—SARS-CoV-2 receptor binding domain (RBD), spike specific monoclonal antibody CR3022	[97]
					*N.benthamiana*—mAbs B38 and H4 neutralizing SARS-CoV-2	[98]
					*N. benthamiana*—ΔXT/FT—cooexpression of component proteins of Human IgA Isotypes	[90, 101]
					*N. benthamiana*—hepatitis B core antigen (HBcAg)	[81]
					*N. tabacum, L. cylindrica*—codon optimised human deoxiribonuclease I; (Dnase I)	[54]
					*N. benthamiana*—GRFT, SP-D, CV-N, hMBL, galectin-9	[92]
					*N. benthamiana*—the recombinant sIgA1 was either expressed alone or co-infiltrated with the vectors carrying genes encoding the proteins for *N*-glycan modification or mucin-type *O*-glycosylation	[56]
Plant biotechnology, nanobiotechnology	Generation of nanobionic plants to improve their natural functions or give new properties	Introducing a suspension of nanoparticles to the target plants.	The specially prepared nanomolecules resuspended in infiltration medium. Common components: MES (10 mM) or TES (10 mM), MgCl_2_ (10 mM) and surfactants, e.g., Silwet L-77 (0.05%)	Spontanaceous infiltration, forced infiltration/whole leaves on the mother plants	*S. oleracea, O. sativa, Pteris cretica*—SWINT-based optical nanosensors	[106]
					*Gossypium hirsutum* L. var. Xinluzao 74, XLZ 74 - poly acrylic acid coated nanoceria; PNC	[27]
					*S. oleracea, Blitum capitatum, L. sativa, Rumex acetosa, Eruca sativa, A. thaliana*—SWNT-based optical nanosensors	[105]
					*A. thaliana*—cerium oxide nanoparticles; nanoceria	[107]
					*A. thaliana*—TGA-QD	[104]

^a^For microbiology references, the studied microorganisms names are listed; for references describing transgenic plant modifications, the studied genes or recombinant proteins are listed; for nanobiotechnology references the used nanomolecules are listed

^b^With particular emphasis on the most recent publications

*AWF* apoplast washing fluid; *AzaC* 5-azacytydine; *C4H* cinnamate-4-hydroxylase; *CBL1* calcineurin B-like calcium sensor protein 1; *CV-N* cyanovirin-N; *GFP* green fluorescent protein; *GT47C* glycosyl-transferase family 47; *Nt FAD2-2* microsomal Δ12 oleate desaturase (1-acyl-2-oleoyl-snglycero-3-phosphocholine Δ12 desaturase); *GRFT* griffithsin; *GUS* beta-glucuronidase; *Hmbl* human mannose-binding lectin; *IgA* immunoglobulin A; *IgG* immunoglobulin G; *LUC* firefly luciferase; *MES* 2-(*N*-morpholino)ethanesulphonic acid; *MtLAP1 M. truncatula* legume anthocyanin producition 1; *MTP1* metal-tolerance protein 1; *PDS* phytoene desaturase; *Prxq* chloroplastic peroxiredoxin Q; *SP-D* surfactant protein D; *SWINT* single-walled carbon nanotubes; *TES* 2-[(2-Hydroxy-1,1-bis(hydroxymethyl)ethyl)amino]ethanesulfonic acid; *TGA-QD* thioglycolic acid quantum dots

## Future prospects

A well-performed infiltration technique (one which has high repeatability) is the basis for obtaining high-quality results in the output procedures. Therefore, the knowledge on the possibilities offered by infiltration and on the impact of different parameters on its effectiveness is crucial for the proper design of the experiment. Leaf infiltration methodology is widely used, but certainly not all infiltration possibilities have been discovered and used so far. Presumably, interest in effective leaf infiltration techniques will grow in the future. Especially agroinfiltration is considered to be a technology that will allow to establish plant bioreactors for efficient production of recombinant proteins used for therapeutic purposes and as vaccines. Recent developments in this field suggest that agroinfiltration-based molecular farming may be of particular importance in the future for the rapid and effective control of a pandemic of new, as yet unknown diseases.

For this kind of future use and in the context of the examples presented in this review it seems interesting and necessary to construct specialized infiltration equipment that would combine the advantages of syringe and vacuum infiltration. The design of such equipment and the optimization of its functions could largely be based on the results described so far [1, 9, 10, 14, 28, 65, 108]. Such a device should (1) be handy to use as a syringe, (2) allow convenient infiltration of even large leaves without the need to detach them from parent plants and cut them into pieces, (3) be small enough and easy to transport, which would enable its in field works, for example for infiltrating plants in their natural growth conditions, (4) generate minimal stress in the target plants. On the other hand, it is important that the infiltration with such equipment can be automated, while allowing for precise control of infiltration parameters. It would also be interesting to provide the leaf infiltration equipment with an additional function enabling the recovery of the infiltrated medium together with the apoplastic fluid (AWF) washed out from the intercellular spaces. The availability of this type of equipment would significantly increase the repeatability of experiments, which include infiltration both as an input and output method.

## Data Availability

Not applicable.

## Abbreviations

AWF: Apoplast washing fluid
AzaC: 5-Azacytydine
C4H: Cinnamate-4-Hydroxylase
CBL1: Calcineurin B-like Calcium Sensor Protein 1
CV-N: Cyanovirin-N
GFP: Green fluorescent protein
GT47C: Glycosyl-transferase family 47
*Nt* FAD2-2: Microsomal Δ12 oleate desaturase (1-acyl-2-oleoyl-snglycero-3-phosphocholine Δ12 desaturase)
GRFT: Griffithsin
GUS: beta-Glucuronidase
Hmbl: Human mannose-binding lectin
IgA: Immunoglobulin A
IgG: Immunoglobulin G
LUC: Firefly luciferase
MES: 2-(*N*-morpholino)ethanesulphonic acid
MtLAP1: *M. truncatula* Legume anthocyanin producition 1
MTP1: Metal-tolerance protein 1
PDS: Phytoene desaturase
Prxq: Chloroplastic peroxiredoxin Q
SP-D: Surfactant protein D
SWINT: Single-walled carbon nanotubes
TES: 2-[(2-Hydroxy-1,1-bis(hydroxymethyl)ethyl)amino]ethanesulfonic acid
TGA-QD: Thioglycolic acid quantum dots
